# Senescence in dahlia flowers is regulated by a complex interplay between flower age and floret position

**DOI:** 10.3389/fpls.2022.1085933

**Published:** 2023-01-13

**Authors:** Matthew Casey, Ilaria Marchioni, Bianca Lear, Alex P. Cort, Ashley Baldwin, Hilary J. Rogers, Anthony D. Stead

**Affiliations:** ^1^ School of Biological Sciences, Royal Holloway University of London, Egham, Surrey, United Kingdom; ^2^ School of Biosciences, Cardiff University, Cardiff, United Kingdom; ^3^ Dipartimento di Scienze Agrarie, Alimentari e Agro-alimentari, Università di Pisa, Pisa, Italy

**Keywords:** Asteraceae, Compositae, cytokinin, *Dahlia pinnata*, ethylene, floral senescence, transcriptome

## Abstract

Mechanisms regulating flower senescence are not fully understood in any species and are particularly complex in composite flowers. Dahlia (*Dahlia pinnata* Cav.) florets develop sequentially, hence each composite flower head includes florets of different developmental stages as the whole flower head ages. Moreover, the wide range of available cultivars enables assessment of intraspecific variation. Transcriptomes were compared amongst inner (younger) and outer (older) florets of two flower head ages to assess the effect of floret vs. flower head ageing. More gene expression, including ethylene and cytokinin pathway expression changed between inner and outer florets of older flower heads than between inner florets of younger and older flower heads. Additionally, based on Arabidopsis network analysis, different patterns of co-expressed ethylene response genes were elicited. This suggests that changes occur in young inner florets as the whole flower head ages that are different to ageing florets within a flower head. In some species floral senescence is orchestrated by the plant growth regulator ethylene. However, there is both inter and intra-species variation in its importance. There is a lack of conclusive data regarding ethylene sensitivity in dahlia. Speed of senescence progression, effects of ethylene signalling perturbation, and patterns of ethylene biosynthesis gene expression differed across three dahlia cultivars (‘Sylvia’, ‘Karma Prospero’ and ‘Onesta’) suggesting differences in the role of ethylene in their floral senescence, while effects of exogenous cytokinin were less cultivar-specific.

## Introduction

The flower heads of the dahlia, a valued ornamental flower from the Asteraceae family, are pseudanthia, ‘false flowers’ (Hutchinson, 1964), also sometimes referred to as a capitulum or a composite flower. Dahlia inflorescences thus develop sequentially with the oldest outermost florets developing, expanding, and opening first. Dahlias (*Dahlia* spp.) are of significant interest to the cut flower industry. However, their commercial potential is limited by their short vase life. Thus, understanding the mechanisms of floral senescence in this species is of both fundamental and commercial interest.

During floral senescence, petals are actively degraded for nutrient remobilisation, culminating in a period of programmed cell death (PCD) (Shibuya et al., 2016). Macromolecules, including proteins and starch are broken down and remobilised to sustain the energy demands of maintaining expensive floral organs for nectar production and, following fertilisation, for the developing ovary and seeds (Ashman & Schoen, 1994). The sequence of events in floral senescence is very similar in flowers on the plant or in cut flowers, although a more rapid senescence off the plant has been noted e.g., in lilies (Arrom and Munne-Bosch, 2012). In composite flowers like dahlia, outer florets senesce before inner florets; this is similar to species with flower spikes, such as gladiolus, where the flowers at the bottom of the spike senescence before those nearer the top (Serek et al., 1994). However, it is not known if senescence is triggered floret by floret or if there is a pan-floral signal, i.e. a signal which triggers senescence across the entire capitulum.

The phytohormone ethylene is a key regulator of floral senescence in many species, associated with pollination (Iqbal et al., 2017; Ma et al., 2018). Ethylene production in these species is regulated auto-catalytically with an initial ethylene burst triggering transcriptional activation of ethylene biosynthetic genes (Ma et al., 2018). Ethylene biosynthesis requires conversion of S-adenosylmethionine (SAM) to 1-aminocyclopropane-1-carboxylic acid (ACC) catalysed by 1-aminocyclopropane-1-carboxylic acid synthase (ACS). The ACC is then oxidised to ethylene by the action of aminocyclopropane-1-carboxylic acid oxidase (ACO). Both enzymes are transcriptionally regulated in both an ethylene-dependent and independent manner in carnation, and transcriptional activation correlates with accelerated senescence across different cultivars (Tanase et al., 2015). Responsiveness to ethylene varies widely across species: Asteraceae species tested including *Chrysanthemum* spp., sunflower (*Helianthus annuus* L.) and dahlia were considered as having very low ethylene sensitivity, while carnation is highly sensitive (Woltering & van Doorn, 1988). Indeed, although treatment with ethylene biosynthesis inhibitors reduced ethylene production in sunflowers, this did not correspond to an improvement in vase life (Mensuali-Sodi & Ferrante, 2005). Moreover, sensitivity can vary across different varieties and cultivars of single species (e.g. Woltering et al., 1993; Wagstaff et al., 2005). The role of ethylene in dahlia senescence remains unresolved: cv. ‘Karma Thalia’ dahlias were unaffected by 16 h exposure to 1 μL L^−1^ ethylene (Dole et al., 2009), or by treatments with STS or 1-MCP (1-methylcyclopropene), inhibitors of ethylene action. However, cut dahlia flowers of cv. ‘Kokucho’ treated continuously with 1 μL L^−1^ 2-chloroethylphosphonic acid (CEPA) solution, which generates ethylene, wilted earlier than those treated with distilled water or citric acid (Shimizu-Yumoto & Ichimura, 2013). Ethylene responses also modulate sensitivity to ethylene during floral senescence (Ma et al., 2018) and the pattern of ethylene receptor expression can vary amongst varieties (Tan et al., 2006). Downstream of the receptor, the large family of ERF transcription factors are important regulators of petal senescence (Chen et al., 2011; Liu et al., 2011) and several ERF genes, especially group VII members are ethylene-regulated.

In contrast to ethylene, exogenous treatment with cytokinins is consistently associated with prolonged flower life across a range of ornamental species such as petunias (Chang et al., 2003), iris (van Doorn et al., 2013), wallflowers (*Erysimum* spp.; Mohd Salleh et al., 2016) and the dahlia’s close relative chrysanthemum (Guo et al., 2003). In dahlias, treatment with cytokinins also seems to be effective in delaying flower senescence in a range of cultivars tested (Shimizu-Yumoto & Ichimura, 2013; Casey et al., 2019) and may act to increase acid invertase activity and sugars (Shimizu-Yumoto et al., 2020). Moreover, inhibiting the degradation of endogenous cytokinin levels also delays floral senescence in carnations (Taverner et al., 2000) and wallflowers (Mohd Salleh et al., 2016), as did increasing endogenous biosynthesis of cytokinins through transformation of petunias (Chang et al., 2003). Petunias transformed to express the cytokinin biosynthetic gene ipt, which encodes the enzyme isopentenyl transferase, placed under the control of the promoter from the senescence associated gene *SAG12*, resulted in flower wilting in transformed plants occurring 6-10 days after control plants (Chang et al., 2003). This suggests that loss of cytokinin is a consistent feature of petal senescence and that increased levels of endogenous cytokinins during senescence can delay the process (Chang et al., 2003).

Cytokinin is sensed by receptors Arabidopsis Histidine Kinases (AHKs) Kieber & Schaller, 2014) and signalling is transduced by Arabidopsis Response Regulators (ARRs). Type-A ARRs are associated with negative feedback of cytokinin regulation and serve to de-sensitise the tissue to cytokinins, whereas type-B ARRs are involved in the transcriptional output of cytokinin perception (Kieber & Schaller, 2014). The type-B ARR, *ARR2*, has been implicated in senescence as its overexpression has been shown to extend leaf longevity in Arabidopsis (Kim et al., 2006). However other type-B ARRs may also be implicated, since double mutants of *ARR1* and *ARR12* show increased dark-induced senescence (Chevalier et al., 2008). Type A-ARRs such as ARR6 (Hallmark and Rashotte, 2020) are up regulated by cytokinin, and type-A ARR mutants also show delayed dark-induced senescence (Li et al., 2012).

A number of recent studies have used RNA-sequencing to study floral senescence in ornamentals, investigating the effect of exogenous cytokinins in petunia (Trivellini et al., 2015), gene expression changes in ethylene insensitive species such as *Gardenia jasminoides* (Tsanakas et al., 2014), and the role of auxins in tepal senescence and abscission in lily (Lombardi et al., 2015). RNAseq has also been used to understand mechanisms underlying pollination-induced corolla senescence in petunia (Broderick et al., 2014; Wang et al., 2018). However, recent transcriptomic studies of Asteraceae species, notably gerbera (Huang et al., 2017), chrysanthemum (Wang et al., 2013; Liu et al., 2016; Won et al., 2017) and sunflower (Liang et al., 2017), have not focused specifically on floral senescence. The few transcriptomic studies in dahlia to date have focused on a comparison of gene expression in different organs (stem, leaf, and flower bud) or on floral buds alone rather than on senescing florets (Hodgins et al., 2014; Lehnert & Walbot, 2014).

Here we present a transcriptomic analysis of dahlia floret senescence revealing that floret senescence is associated with changes in gene expression both within a capitulum and between capitula. This suggests a complex mechanism regulating senescence progression, of relevance to other species with composite flower heads. A detailed analysis of floret senescence on and off the plant, and responses to ethylene and cytokinin treatments, confirms variation amongst dahlia cultivars both at a physiological and gene expression level.

## 2 Materials and methods

### 2.1 Plant material growth and collection

Tubers of *Dahlia pinnata* cultivars ‘Sylvia’, ‘Onesta’ and ‘Karma Prospero’ were purchased from ‘Rose Cottage Plants’ (Essex, UK). In the 2015 growing season the RHS Wisley research site (Deer Farm, Wisley, Surrey, UK) was used and in the 2016 and 2017 seasons dahlias were planted at Royal Holloway University of London (Egham, Surrey, UK). Dahlias were potted in multi-purpose peat-based compost (Longacres, Bagshot, UK) and grown in pots in a poly-tunnel until late May before being planted outside for the remainder of the growing season with the addition of appropriate fertiliser (20% N, 20% P, and 20% K with micronutrients including Mn and trace elements; ‘Peters Professional Allrounder’). Material for RNA-sequencing was collected during the 2015 and 2016 growing seasons, and material for postharvest treatments and PCR was collected during the 2017 growing season.

Once harvested, flowers were placed in tap water, at a constant temperature room set to 21°C and a 12 h photoperiod from cool white fluorescent tubes (15-20 µM m^-2^ sec-^1^). All leaves were removed, and stems cut to lengths of 5 cm. Development of flowers was divided into five stages (**Supplementary Figure S1**). At stage I just the outer whorl of florets have opened and they are at no more than a 45° angle to the innermost florets. Stage II inflorescences have outermost florets that have progressed to a 90° angle compared to the innermost florets. By stage III the green innermost undeveloped florets have begun to become more obscured compared to stage I and II inflorescences, and the outermost florets are at a >90° angle compared to innermost florets but have not begun to curl back. At stage IV the outer florets have opened at a 180° angle compared to the innermost florets. By stage V the flower has fully opened, in ‘Sylvia’ forming the distinct ‘ball’ type dahlia, with the outermost florets curled back so far as to obscure the receptacle and almost no green developing inner florets can be seen. The developmental stages outlined are standard in the horticultural sector, and such staging is broadly modelled on that used by the Dutch Flower Auctions Association. Days between stages are: Stage I-II: 2 days, II-III: 2 days, III-IV: 1.5 days and IV-V: 1.5 days. However, note that plants were grown outdoors (to mimic commercial conditions) and the time taken to reach different stages can vary depending on weather conditions.

### 2.2 Postharvest treatments

Postharvest treatments were based on previous work (Dole et al., 2009; Shimizu-Yumoto & Ichimura, 2013) and consisted of 6-benzylaminopurine (BA, 0.1 mM), ethephon (chloroethylphosphonic acid or CEPA, 0.02 mM) and silver thiosulphate (STS, 4 mM) all dissolved in distilled water (dH_2_O). Control treatments were dH_2_O as a vase solution or spray. Five replicate flowers were used for each treatment. Treatments were applied continuously from harvest, pulsed in the vase water for a specified time period and then placed in distilled water, or sprayed. Flowers were sprayed from a distance of 30 cm in a fume hood until the solution had been applied to the whole flower surface and left to dry.

Flowers were harvested at stage III (**Supplementary Figure S1**) for vase life trials (Armitage & Laushman, 2003). Flowers selected had no visible disc florets bearing pollen, and therefore were very unlikely to have been pollinated. Vase life was considered finished when the outer two whorls of florets showed visible signs of senescence. Signs of senescence include petal wilting, curling, discolouration, and abscission (van Doorn & Woltering, 2008).

Statistical analysis for all physiological assays following postharvest treatments used 2-way ANOVA followed by a Tukey’s test, or if data did not conform to normality and equal variance, a non-parametric test: Kruskal Wallis followed by a Dunn’s test. Tests were carried out using RStudio Desktop (version 1.1) on R (version 3.5).

### 2.3 Floret mass and conductivity measurements

Flowers were harvested at stage III (**Supplementary Figure S1**). Floret mass was measured 1, 4, and 7 d after harvest. The mean weight of six florets (weighed individually) from the middle whorl of each of five inflorescences were used for each replicate at each time point. A different group of five flowers was used for each time point. After weighing, pairs of florets from each replicate flower head were placed in 15 ml of dH_2_O and the conductivity of the solution measured using an Accumet AP75 data meter (Fisher Scientific) after 3 h. Conductivity was measured again after autoclaving to express it as a percentage of total conductivity of petals (Whitlow et al., 1992). The average of the three floret pairs from each flower was considered a biological replicate.

### 2.4 RT-qPCR following postharvest treatments

Flowers were harvested at stage III in line with the postharvest treatment flowers (**Supplementary Figure S1**). For RT-qPCR, NucleoSpin^®^ RNA Plant (Macherey-Nagel) was used for RNA extraction. For treated flowers, RNA for each biological replicate was extracted from a ground mix of ten middle whorl florets from five flowers, divided randomly into three groups. Florets from the same five whole flowers were used for RNA extraction mass and conductivity measurements. In both cases a maximum of 100 mg floret tissue (sexual organs were removed) was ground in liquid nitrogen and the protocol was performed according to the manufacturer’s instructions. Residual genomic DNA was eliminated using gDNA Wipeout buffer from the Quantitect Reverse Transcription kit (QIAGEN) according to the manufacturer’s protocol. cDNA was prepared using 0.5 µg of RNA with a Quantitect Reverse Transcription kit (QIAGEN) according to the manufacturer’s protocol.

qRT-PCR was carried out according to a Rotor-Gene SYBR Green PCR Kit protocol (QIAGEN) and using a Rotor-Gene Q qPCR machine (QIAGEN). Three biological replicates and three technical replicates were used for each sample. β-tubulin was used as a reference gene due to its Ct values being much closer on average to target genes compared to 18S rRNA. The relative levels of expression were calculated using the formula from Pfaffl (2001). All primers for qRT-PCR are listed in **Supplementary Table S1**.

### 2.5 RNA-sequencing and RT-qPCR verification

For RNA sequencing (RNAseq) and subsequent RT-qPCR, three stages of cv. ‘Sylvia’ florets were used: inner stage III florets (SIII-in) and both inner and outer stage IV florets (SIV-in, and SIV-out) (**Supplementary Figure S1**). Three biological replicates for each developmental stage were obtained by mixing nine florets from the same stage of three flower heads and dividing into three random groups. Florets for each replicate were ground under liquid nitrogen and RNA extracted using an RNEasy Plant MiniKit (QIAGEN) for RNAseq or according to Gambino et al. (2008) for RT-qPCR. RNA for RNAseq was quality tested using a Qubit fluorometer and samples were prepared with a Truseq Illumina stranded mRNA kit, normalised to equimolar ratios and then sequenced using an Illumina NextSeq 500 to produce paired-end 150 nt reads for each sample. For the first of the three replicates of each group a higher read depth of sequencing was opted for to ensure a greater coverage of the transcriptome and to give a greater chance of finding rare transcripts. Quality control was performed using the FastQC tool (version 0.11.5) to assess base and sequence quality (Andrews, 2010). Transcriptome sequence reads have been deposited in the Sequence Read Archive (SRA) database at NCBI (BioProject ID: PRJNA742864). Subsequent RT-qPCR was as above except a SYBR Green PCR Kit (PCR Biosystems) in a total volume of 10 µl (4 µL of cDNA and 6 µL of SYBR Green) and a Light cycler qPCR machine (Roche) were used.

### 2.6 *De novo* assembly and analysis of transcriptome

Forward and reverse reads from all the samples were assembled into a reference transcriptome using the Trinity software package (version 2.3.2) with default settings (Grabherr et al., 2011), comprising Inchworm that assembles reads into unique transcripts, Chrysalis that clusters transcripts and constructs de Bruijn graphs for each cluster and Butterfly that processes the de Bruijn graphs into full length transcripts, including transcripts for alternatively spliced isoforms and paralogous genes. Trimmomatic (version 0.35) was used to remove low quality reads, low quality bases (including N bases) and adapter regions from the data (Bolger et al., 2014). The Galaxy online platform (Galaxy version 0.2.01) was used to map reads from each sample onto the Trinity reference transcriptome, using the TopHat2 alignment software on default settings.

Blastx alignment queries against the translated *Arabidopsis thaliana* genome (TAIR10; Lamesch et al., 2012; EnsemblPlants, 2018) were performed using blastx (Galaxy version 0.2.01) on the Galaxy online platform (Camacho et al., 2009; Cock et al., 2015; Afgan et al., 2018) using default settings and an e-value equal to or less than 1e^-5^, as used previously (Lehnert & Walbot, 2014). Alignments with a bit score of less than 50 were removed (Pearson, 2013).

### 2.7 Differential expression and functional analysis

Mapped reads were inputted into the Cufflinks pipeline for differential expression analysis (Galaxy Version 2.1.1) (Kim et al., 2013; Afgan et al., 2018) using: Cufflinks (Galaxy version 2.2.1.2), Cuffmerge (Galaxy 2.2.1.1 version) and Cuffdiff (Galaxy version 2.2.1.5) (Trapnell et al., 2010). Cufflinks was used to assemble and estimate the abundance of the aligned reads generated using TopHat2 and was performed using the *Helianthus annuus* annotation (EnsemblPlants, 2018) as a guide and otherwise default settings (Badouin et al., 2017). Pathway analysis was carried out using KEGG (Kanehisa & Goto, 2000). Differences in differentially expressed gene (DEG) gene ontology (GO) annotations were assessed using http://www.pantherdb.org (Mi et al., 2019). Singular Enrichment Analysis (SEA) by agriGO followed by GeneMANIA (Multiple Association Network Integration Algorithm; Mostafavi et al., 2008) within Cytoscape v3.8.2 was used to construct predicted co-expression maps of DEGs related to ethylene signalling based on annotation to the *Arabidopsis thaliana* genome.

## 3 Results

### 3.1 Dahlia floret senescence progressed more rapidly in cut flowers compared to on the plant

**Figure 1:**
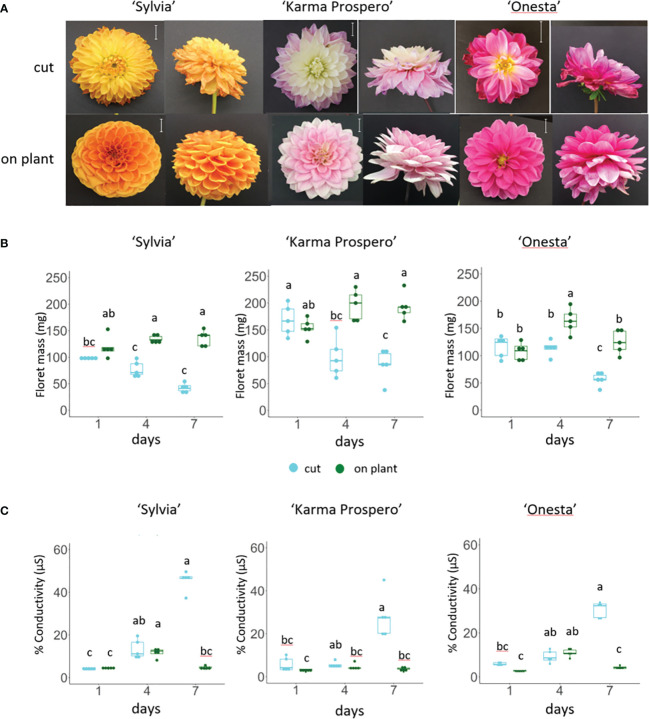
Floret senescence in dahlia cv.s Sylvia, Karma Prospero and Onesta in response to cutting from the plant **(A)** flower head appearance 7 days after harvesting at Stage III: cut flower stems were held in distilled water, compared to uncut flowers left on the plant; (scale bars represent 20 mm); **(B)** floret mass **(C)** ion leakage, in mid-whorl florets 1, 4 and 7 days after cutting stage (n=5). Different letters indicate significant differences *p* < 0.05, based on a 2-way ANOVA followed by a Tukey’s test or a Kruskall Wallis test followed by a Dunn’s *post hoc* test if the data did not fit the normality and equal variance criteria required.

To investigate dahlia floral senescence progression, three cultivars, ‘Sylvia’, ‘Karma Prospero’ and ‘Onesta’ were assessed for floret senescence progression on the plant and when harvested at stage III (**Supplementary Figure S1**) held in distilled water. All three cultivars showed visible flower deterioration after 7 days as cut flowers (wilting, curling and or discolouration), whereas on the plant there was little visible deterioration (**Figure 1A**). In addition, both ‘Sylvia’ and ‘Onesta’ cut flowers opened less compared to uncut flowers. Overall, there was a significant interaction between time and treatment for the change in mass in all three cultivars (*p* < 0.001). By day 4, in cut flowers there was a significant (*p* < 0.05) reduction in floret mass compared to uncut in all three cultivars (**Figure 1B**), but the reduction in fresh weight at day 7 was only significantly lower than at day 4 in cut flowers in cv. ‘Onesta’. Ion leakage, a measure of cell death, increased in cut flowers post-harvest, with a significant (*p* < 0.001) interaction between time and treatment in all three cultivars. The increase in ion leakage in cut flowers was greatest at day 7 where it was significantly higher (over 7-fold) than the uncut controls (*p* < 0.05) in all three cultivars (**Figure 1C**). In contrast, although ion leakage increased by day 4 on plant in cv. ‘Sylvia’ (by 2.5-fold) and ‘Onesta’ (by 4-fold) flowers it did not increase significantly over this time period in cv. ‘Karma Prospero’ flowers on the plant. Thus, there was progressive floret deterioration off the plant in all three cultivars, with some intra-specific variation.

### 3.2 Transcriptome sequencing and *de novo* assembly

To investigate mechanisms of senescence in dahlia florets, RNAseq analysis was employed comparing gene expression in cv. Sylvia. Younger and older florets in the same flower head (inner (SIV-in) and outer (SIV-out) florets of stage IV flowers and the florets from the same relative position, in heads of differing age: stage III (SIII-in) and stage IV (SIV-in) flowers were compared, as well as the extremes (SIII-in and SIV-out). (**Supplementary Figure S1**). Across all nine samples (three replicates of each floret stage) a total of 345,038,365 reads were generated. The mean sequence quality of reads for each sample was >34 indicating high quality (Andrews, 2010; Babraham Bioinformatics, 2018). Given the lack of a dahlia genome sequence, *de novo* assembly using Trinity was used to generate 289,538 contigs, which were further reduced using TopHat2 to 137,376 contigs of high enough quality to be successfully mapped using the *de novo* assembly produced by Trinity.

### 3.3 Overall patterns of differential expression analysis

**Figure 2:**
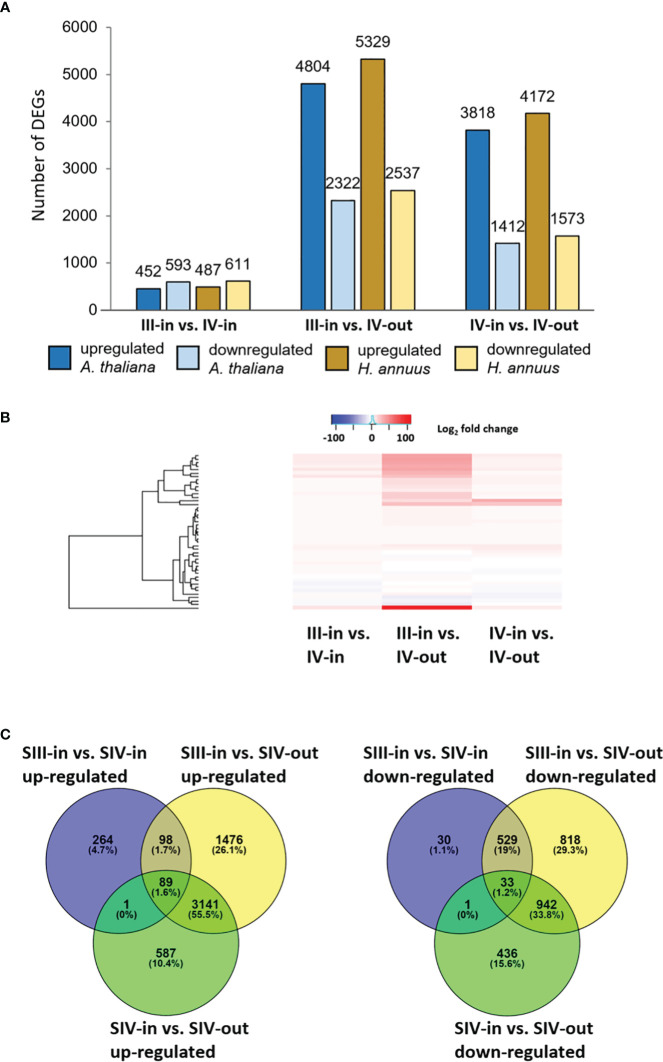
Transcriptomic analysis of dahlia florets **(A)** Number of DEGs homologous to *A thaliana* or *H annuus* proteins in each sample comparison; **(B)** heat map of up and downregulated genes, and present in all sample comparisons. Red to blue scale shows Log_2_ fold change (*p. adjust* < 0.05); **(C)** Venn diagrams of up- and down regulated genes, based on annotation to *Arabidopsis thaliana* (*p adjust.* < 0.05) inner florets of Stage III flowers (SIII-in), inner florets of Stage IV flowers (SIV-i) and outer florets of Stage IV flowers (SIV-out).

**Figure 3:**
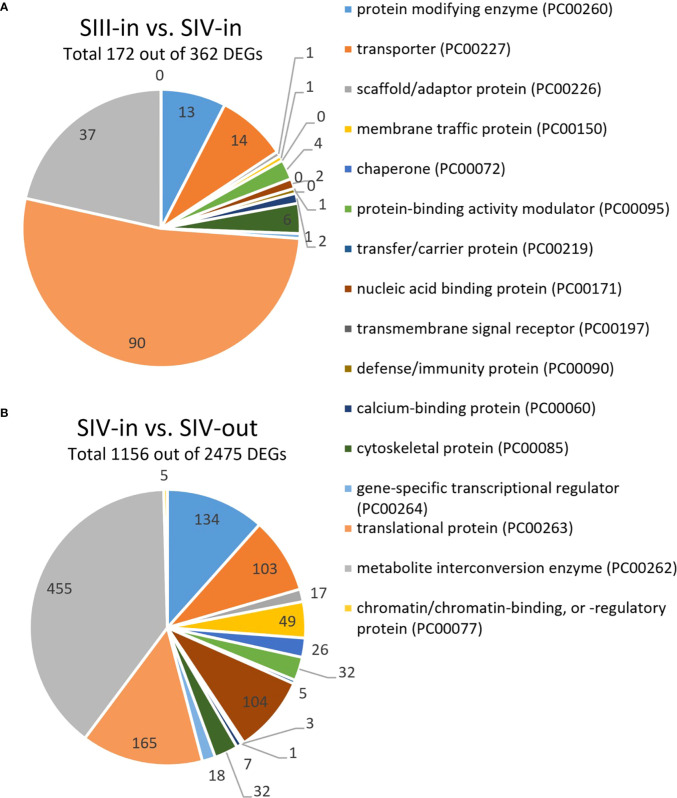
Most notable differences in GO annotations for DEGs from transcriptomic analysis of dahlia florets based on annotation to *Arabidopsis thaliana* (*p adjust.* < 0.05) **(A)** inner florets of Stage III flowers (SIII-in) vs. inner florets of Stage IV flowers (SIV-i) and **(B)** inner florets of Stage IV flowers (SIV-i) vs. outer florets of Stage IV flowers (IV-out). GO annotation based on http://www.pantherdb.org; Mi et al. (2019).

Between 1.9% and 11.5% of the contigs showed differential expression between the floret stages. Approximately half of these differentially expressed genes (DEGs) could be annotated using Blast X, with slightly more annotated when compared to *Helianthus annuus* (14 709) than to *Arabidopsis thaliana* (13 401; **Figure 2A**). However, annotation to Arabidopsis genes allowed access to more bioinformatics tools and was hence used for further analysis. Overall, more floret genes changed in expression with position in the flower head (SIV-in vs. SIV-out) than with head age (SIII-in vs. SIV-in), and most changes occurred between the extremes (SIII-in and SIV-out). In both the comparisons between SIII-in vs. SIV-out and SIV-in vs. SIV-out florets, more genes were up regulated than down regulated indicating an active process. In contrast slightly more genes were down regulated in the comparison between SIII-in and SIV-in florets. Overall, the positive fold change in up regulated genes was greater than the negative fold change in the down regulated genes (**Figure 2B**), with greatest log_2_ fold changes in the SIII-in vs. SIV-out comparison, and blocks of genes showing significant log_2_ fold change in all three comparisons. A more detailed comparison of the overlaps in gene expression patterns based on annotation to *Arabidopsis thaliana* sequences shows that a far greater number of DEGs were shared between SIII-in vs. SIV-out florets and SIV-in vs. SIV-out florets both amongst up regulated (3230, 57%) and down regulated (975, 35%) than with SIII-in vs. SIV-in. Just 122 DEGs (89 up and 33 down regulated DEGs) were common to all floret stage comparisons (**Figure 2C**). An overall analysis of GO annotations comparing the effects of position in the head or head age show that although similar functional classes are represented, their relative proportions differ (**Figure 3**). For example, the largest proportion of the genes are assigned to translational proteins in DEGs between head ages, while the largest proportion of genes are assigned to metabolite interconversion and nucleic acid binding functions in DEGs between head positions.

### 3.4 Cell death associated genes are up regulated with dahlia floret age, while senescence, autophagy, vacuolar processing enzyme, caspase and metacaspase genes are both up and down regulated

**Figure 4:**
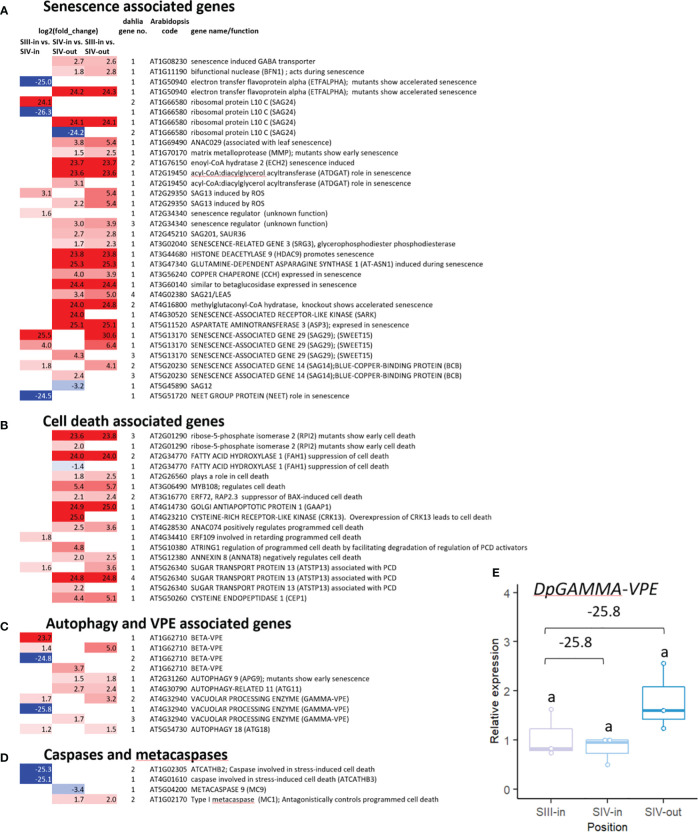
Transcriptome DEGs associated with **(A)** senescence **(B)** cell death **(C)** autophagy and vacuolar processing enzymes (VPEs), **(D)** caspases and metacaspases. Red to blue scale shows Log_2_ fold change (*p. adjust* < 0.05); based on annotation to *Arabidopsis thaliana;* inner florets of Stage III flowers (III-in), inner florets of Stage IV flowers (IV-i) and outer florets of Stage IV flowers (IV-out); gene no. indicates number of dahlia genes with homology to each Arabidopsis gene with similar expression pattern. **(E)** RT-qPCR of *Dp*γ-VPE expression, n=3, different letters indicate significant differences *p* < 0.05, based on a 2-way ANOVA followed by a Tukey’s test or a Kruskall Wallis test followed by a Dunn’s *post hoc* test if the data did not fit the normality and equal variance criteria required. RNA seq log2FC shown on graph.

The majority of senescence-associated dahlia genes (42 out of 48) were up regulated either in the SIII-in vs. SIV-in or SIV-in vs. SIV-out comparisons, 15 of them by >20 log_2_ FC (**Figure 4A**; **Supplementary Table 2A**). Fewer senescence genes changed in expression between florets of different head age, compared to floret position. For several genes, different dahlia genes matching the same Arabidopsis gene showed strongly contrasting expression patters e.g. *DpETFALPHA* and *DpSAG24* (ribosomal protein LC10). Different dahlia orthologues of *SAG13*, which in Arabidopsis is expressed early in senescence, were up regulated by 2-3 fold in one but not the other floret comparison, however *DpSAG21/LEA5* which is also expressed early and transiently in senescence was only up regulated in the comparison between floret position in the same older SIV flower head. *DpSAG12*, considered a marker of late senescence was in fact down regulated in this comparison.

Twenty-four genes whose function is associated with cell death processes, including homologous genes to the sugar transporter *ATSTP13*, and the cysteine endopeptidase *CEP1* were all up regulated in one of the two floret comparisons (**Figure 4B**; **Supplementary Table 2A**). More DEGs with homology both to autophagy and vacuolar processing enzymes (VPEs) (**Figure 4C**) as well as caspases and metacaspases (**Figure 4D**) changed in relation to flower head age than in relation to position in the head. Twelve dahlia DEGs showed homology to VPEs, six beta and six gamma, but expression of different members of the gene family contrasted strongly in expression being both strongly up and down regulated. All three autophagy related genes were up regulated but only by 1.2-2.7 log_2_ FC, with dahlia orthologues of *APG9* and *ATG11* genes up regulated in relation to floret position, and a dahlia *ATG18* in relation to flower head age, although weakly. Two dahlia caspase genes (similar to *ATCATHB2* and *ATCATHB3*) were strongly down regulated in relation to increasing head age, while amongst the metacaspase-like genes the dahlia *MC9* gene was down regulated and the dahlia *MC1* gene was up regulated in relation to floret position.

A single dahlia contig with log_2_ FC of -25.8 between florets of similar age acorss different aged heads (SIII-in vs. SIV-in) showed homology to γ-VPE (TCONS_00133099; **Supplementary Table 2A**). Its RNAseq expression pattern was assessed using RT-qPCR (**Figure 4E**). There was a slight down regulation between SIII-in and SIV-in florets although due to the variability the change was not statistically significant. In contrast to the RNAseq data, RT-qPCR revealed slight upregulation in SIV-out florets compared to both SIII-in and SIV-in florets although again the change was not statistically significant probably due to the variability in expression.

### 3.5 Expression patterns of transcription factors differ comparing floret position in flower head and florets in different ages of flower head

**Figure 5:**
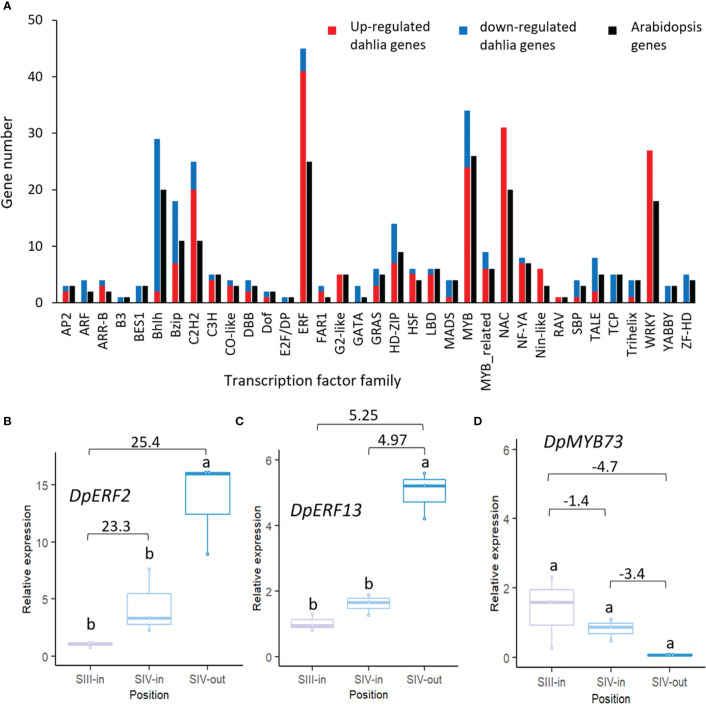
Transcription factors differentially expressed in at least one dahlia floret stage comparison. **(A)** Numbers of genes of each family amongst the DEGs in the dahlia transcriptome and number of nearest Arabidopsis gene homology match. Red and blue indicate numbers of genes in each family that were all up or down regulated, respectively; **(B–D)** RT-qPCR analysis of **(B)** *DpERF2* **(C)** *DpERF13* **(D)** *DpMYB73*, n=3, different letters indicate significant differences *p* < 0.05, based on a 2-way ANOVA followed by a Tukey’s test or a Kruskall Wallis test followed by a Dunn’s *post hoc* test if the data did not fit the normality and equal variance criteria required. RNA seq log2FC shown on graph.

A total of 340 dahlia contigs that were differentially expressed amongst the floret stages showed homology to 232 Arabidopsis transcription factors falling into 35 different families (**Figure 5A**; **Supplementary Table S2B**). Thus, in most families, more than one dahlia contig matched the same Arabidopsis gene. Overall, there were very few changes in expression of dahlia transcription factor genes related to flower head age (SIII-in vs. SIV-in). In all cases up or down regulation was consistent amongst the three different floret comparisons. The highest number of dahlia transcription factors (45) were in the *ERF* family, with the majority (91%) up regulated, twelve genes by > 20 fold log _2_ FC, between SIII-in and SIV-out florets. *MYB* and *NAC* family TFs were also highly represented (34 and 31 dahlia genes respectively); all *NAC* TFs were up regulated with three of them > 20 fold log _2_ FC. In contrast although six *MYB* TFs were strongly (> 20 fold log _2_ FC) up regulated, 29% of the MYB TFs were down regulated. All of the 27 WRKY TFs were also up regulated. The majority of bHLH (93%) were down regulated while 39% of bZIP and 80% of C2H2 TFs were up regulated. In seven TF families (*ARF*, *B3*, *BES*, *E2F/DP*, *GATA*, *YABBY* and *ZF-HD*) all the dahlia genes represented in the DEGs were down regulated in at least one floret comparison and were not up regulated in any of the floret comparisons.

Expression patterns of two *ERF* (*ERF2* and *ERF13*-like) and one *MYB* TF were verified by RT-qPCR. Two dahlia contigs matched Arabidopsis *ERF2* with slightly contrasting expression (**Supplementary Table S2B**) although in both cases there was strong up-regulation between SIII-in and SIV-out florets. RT-qPCR of TCONS_00111354 confirmed that there was some upregulation between SIII-in and SIV-in (though not statistically significant) as well as between SIII-in and SIV-out, but in contrast to the RNAseq there was also upregulation between SIV-in and SIV-out (**Figure 5B**). Of the three dahlia contigs matching *ERF13* (**Supplementary Table S2B**), two were upregulated both between SIII-in and SIV-out, as well as between SIV-in and SIV-out but not SIII-in vs. SIV-in. RT-qPCR of TCONS_00071434 confirmed the strong up-regulation in SIV-out florets compared to the other two stages (**Figure 5C**). A single dahlia contig matched Arabidopsis *MYB73* (TCONS_00133600; (**Supplementary Table S2B**) and RT-qPCR was consistent with the RNAseq data indicating a slight down-regulation both between SIII-in vs SIV-in and SIV-in vs. SIV-out, although not statistically significant in the RT-qPCR (**Figure 5D**).

### 3.6 Differential expression analysis of ethylene and cytokinin related genes

**Figure 6:**
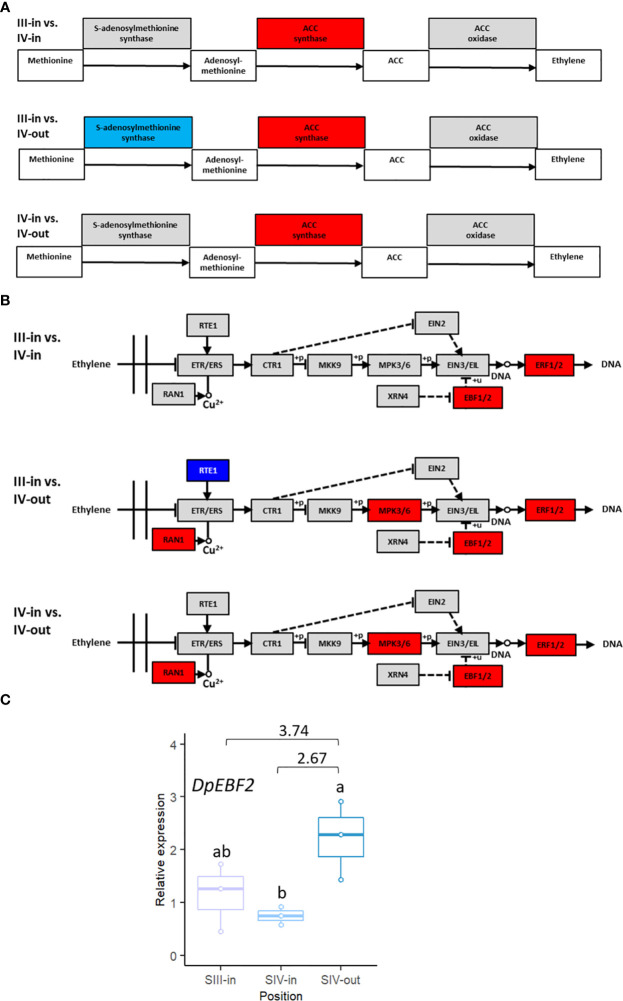
Changes in expression of dahlia floret genes associated with ethylene **(A)** biosynthesis **(B)** signal transduction pathway in comparisons between florets from: SIII-in vs. SIV-in, SIII-in vs. SIV-out and SIV-in vs. SIV-out (from KEGG analysis of RNAseq data, Kanehisa & Goto, 2000). Grey indicates no significant change in gene expression, red indicates significantly upregulated and blue indicates significantly downregulated (*p* < 0.05) **(C)** RT-qPCR of *DpEBF2* expression, n=3, different letters indicate significant differences *p* < 0.05, based on a 2-way ANOVA followed by a Tukey’s test or a Kruskall Wallis test followed by a Dunn’s *post hoc* test if the data did not fit the normality and equal variance criteria required. RNA seq log2FC shown on graph.

Given its known relevance in relation to floral senescence, transcriptome DEGs were analysed to assess changes in ethylene-related gene expression. Based on KEGG analysis of pathways, rate limiting enzyme ACC synthase was up regulated in all three floret stage comparisons. SAM synthase was down regulated in the comparison between SIII-in florets and SIV-out florets but not in the other two floret comparisons (**Figure 6A**), while ACC oxidase expression was unchanged. Genes encoding four enzymes within the ethylene signal transduction pathway, MPK3/6, RAN, ERF1/2 and EBF1/2, were upregulated when SIII-in florets were compared to SIV-out florets or SIV-in and SIV-out florets (**Figure 6B**). In contrast, in the comparison between the SIII-in florets and SIV-in IV florets only genes encoding EBF1/2 and ERF1/2 were up regulated.

In addition to *ERF2* detailed above, expression of *EBF2* was also verified by RT-qPCR. Four *EBF2* genes matched the Arabidopsis gene (**Supplementary Table S2C**). The expression pattern of TCONS_00123767 was in agreement with the RNAseq data: upregulated between SIV-in and SIV-out florets (**Figure 6C**) and between SIII-in and SIV-out although the latter difference was not statistically significant using RT-qPCR.

**Figure 7:**
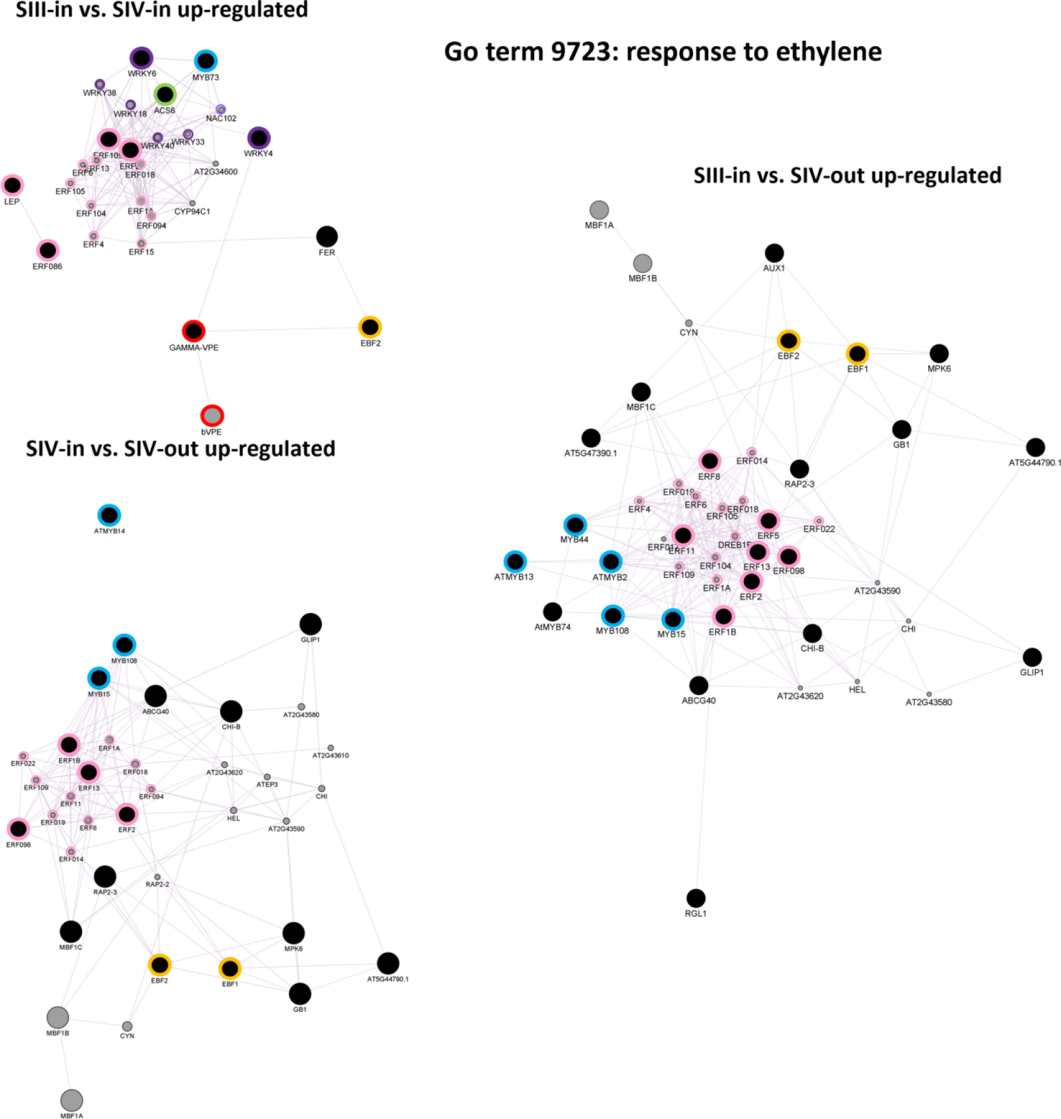
Co-expression gene networks of up-regulated DEGs identified as belonging to the GO term: response to ethylene. Constructed using GeneMANIA within Cytoscape using annotation to *A. thaliana* proteins, for each sample comparison: SIII-in vs. SIV-in, SIII-in vs. SIV-out and SIV-in vs. SIV-out, where stages are inner florets of Stage III flowers (SIII-in), inner florets of Stage IV flowers (SIV-i) and outer florets of Stage IV flowers (SIV-out). Black filled circles indicate dahlia floret DEGs. Grey filled circles indicate genes identified by the software as co-expressed with the Arabidopsis homologue of the dahlia gene, and their circle size is proportional to the number of interactions. Green bordered circles indicate ethylene biosynthesis genes; purple: WRKY and NAC, blue: MYB, pink ERF transcription factors; red, vacuolar processing enzymes, yellow: EIN3-BINDING F BOX proteins.

Ethylene signalling was explored further using singular enrichment analysis based on the annotation to Arabidopsis genes. Given the KEGG analysis showing up regulation of key biosynthetic and ethylene response genes, of most interest were co-expression networks of the up regulated “Response to ethylene” (GO:0009723) DEGs. Clear differences were evident in the three floret stage comparisons (**Figure 7**; **Supplementary Table S3**). Co-expressed genes within the DEGs from the SIII-in and SIV-in floret comparison included *ACS6*, two *WRKY*, one *MYB* and four *ERF* transcription factors (TFs). These in turn formed a network with four other WRKY and nine other ERF TFs. In addition, a co-expression link was also found between expression of WRKY4 and a γ-VPE gene which in turn was also co-expressed with an EIN3-BINDING F BOX protein. In contrast, no WRKY TFs were identified in the co-expression analysis amongst the DEGs from the comparison between SIII-in and SIV-out florets, whereas more MYB TFs (three) and the same number of ERF TFs (but different genes) were present. Vacuolar processing enzymes were not identified in this network, however two EIN3-BINDING F BOX proteins were present. When the SIII-in and SIV-out dahlia floret DEGs were analysed for co-expression networks, even more MYB TFs were identified (five), again no WRKY TFs, but more ERF TFs (seven) with a further eleven ERFs in the network. In this comparison, again both EIN3-BINDING F BOX genes were present.

**Figure 8:**
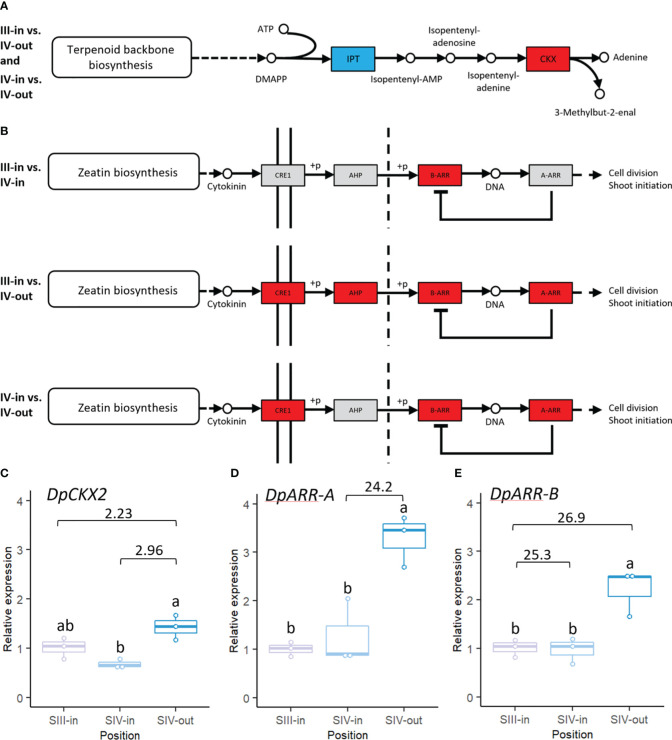
Transcriptomic changes in expression of dahlia floret genes associated with cytokinin **(A)** biosynthesis **(B)** signal transduction pathway in comparisons between florets from: III-in vs. IV-in, III-in vs. IV-out and IV-in vs. IV-out (from KEGG, Kanehisa & Goto, 2000). Grey indicates no significant change in gene expression, red indicates significantly upregulated and blue indicates significantly downregulated (*p* < 0.05). There were no significant changes in expression of cytokinin biosynthesis related genes in III-in vs. IV-in florets. **(C–E)** RT-qPCR analysis of **(C)** *DpCKX2* **(D)** *DpARR-A* **(E)** *DpARR-B*, n=3, different letters indicate significant differences *p* < 0.05, based on a 2-way ANOVA followed by a Tukey’s test or a Kruskall Wallis test followed by a Dunn’s *post hoc* test if the data did not fit the normality and equal variance criteria required. RNA seq log2FC shown on graph.

Also of interest was the representation of cytokinin biosynthesis and signal transduction pathways in the dahlia floret DEGs. In comparisons between SIV-in and SIV-out florets and SIII-in with SIV-out florets cytokinin biosynthetic genes, isopentenyl transferases (IPT’s), were down regulated while cytokinin oxidases implicated in cytokinin catabolism were up regulated (**Figure 8A**). No significant changes in cytokinin oxidases or IPT’s were found in comparison between SIII-in and stage IV florets. Most cytokinin response genes were up regulated in the DEGs from SIII-in florets and SIV-out florets (**Figure 8B**). These included a type-B-ARR that was up regulated in all sample comparisons as well as CRE1 and a type-A ARR which were also up regulated in the comparison between SIV-in and SIV-out florets. AHP was only up regulated in the comparison between SIII-in florets and SIV-out florets. No genes in these pathways were significantly down regulated.

RT-qPCR confirmed the up-regulation of CKX2 (TCONS_00108633) between both SIII-in and SIV-in vs. SIV-out (**Figure 8C**; **Supplementary Table S2C**) although the SIII-in vs. SIV-out comparison was not statistically significant in the RT-qPCR. The upregulation between SIV-in and SIV-out of the dahlia contig with homology to ARR-A (TCONS_00091529) was consistent between the RNAseq and RT-qPCR analysis (**Figure 8D**). Two dahlia contigs showed homology to ARR-B (TCONS_00090228 and TCONS_00090227); RT-qPCR confirmed upregulated expression of TCONS_00090228 between SIII-in and SIV-out, but expression in SIV-in and SIII-in was not significantly different (**Figure 8E**).

### 3.7 Differential responses amongst cultivars to inhibition of ethylene signalling with STS

**Figure 9:**
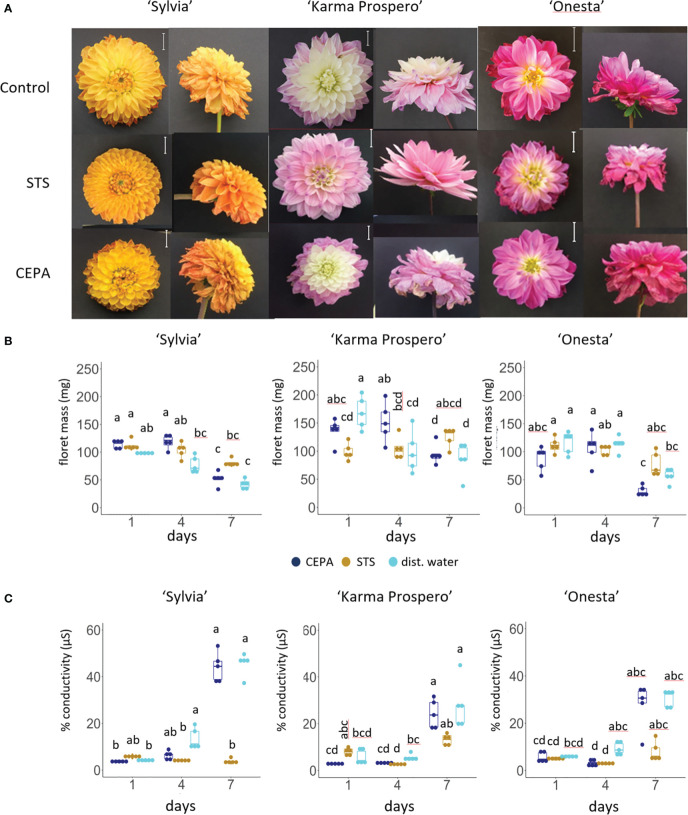
Effect of ethylene signalling perturbation on floret senescence in dahlia cv.s Sylvia, Karma Prospero and Onesta. Stems were held in distilled water (control), compared to stems treated with a 1 h pulse of 4 mM STS or with 20 µM CEPA. **(A)** Flower head appearance 7 days after harvesting at Stage III; scale bars represent 20 mm); **(B)** floret mass **(C)** ion leakage, 1, 4 and 7 days after cutting stage (n=5). Different letters indicate significant differences *p* < 0.05, based on a 2-way ANOVA followed by a Tukey’s test or a Kruskall Wallis test followed by a Dunn’s *post hoc* test if the data did not fit the normality and equal variance criteria required.

Given the changes in expression shown by RNAseq in ethylene signaling during floret senescence, responses to exogenous ethylene and inhibition of ethylene signaling were compared across different cultivars. Symptoms of senescence after 7 days were visibly improved compared to controls by a 1 h pulse with 4 mM STS in both cv. ‘Sylvia’ and ‘Karma Prospero’ but not in cv. ‘Onesta’ (**Figure 9A**). After 7 days, CEPA treated flowers of all three cultivars showed more wilting and floret browning than controls. However, the effects of STS and CEPA on appearance were not reflected in many significant changes in floret mass (**Figure 9B**). There was an interaction between treatments and time for both ‘Sylvia’ (*p* < 0.05) and ‘Karma Prospero’ (*p* < 0.001) but not ‘Onesta’ in relation to floret mass. In cv. ‘Sylvia’ there were few significant differences in fresh weight in response to STS or CEPA across the samples. In ‘Karma Prospero’ floret fresh weight remained more stable post-harvest in STS treated flowers compared to controls where mass fell significantly (*p* < 0.05), by 1.7-fold, between day 1 and day 4. CEPA treatment did not reduce fresh weight any more than in controls. In cv. ‘Onesta’, STS treatment abolished the significant (*p* < 0.05) 2-fold reduction in floret weight between day 1 and day 7, seen in controls, while CEPA increased at the weight loss between day 1 and day 7 to nearly 3-fold, although the loss was not significant due to the variability at day 1. There was an interaction between treatment and time for ion leakage as well (*p* < 0.01) for all three cultivars. In cv. ‘Sylvia’, STS had a dramatic effect on floret ion leakage at day 7 which was 11 fold higher in controls, compared to STS treated florets. There was also a significant (*p* < 0.05) 3-fold reduction in ion leakage at day 4 in STS treated florets compared to the control (**Figure 9C**). In contrast, STS had no significant effect on ion leakage of ‘Karma Prospero’ florets even at day 7 where there was no significant difference (*p* <0.05) between STS treated and control florets. The effect on ‘Onesta’ florets was intermediate with a mean reduction in ion leakage in STS treated florets compared to the controls, which was only statistically significant on day 4. CEPA had very little effect on ion leakage in all three cultivars compared to the control with even a slight but significant reduction at day 4 in cv. ‘Onesta’ florets.

### 3.8 Cytokinin (BA) treatment elicited a strong response in retarding senescence but effects were dependent on method of application

**Figure 10:**
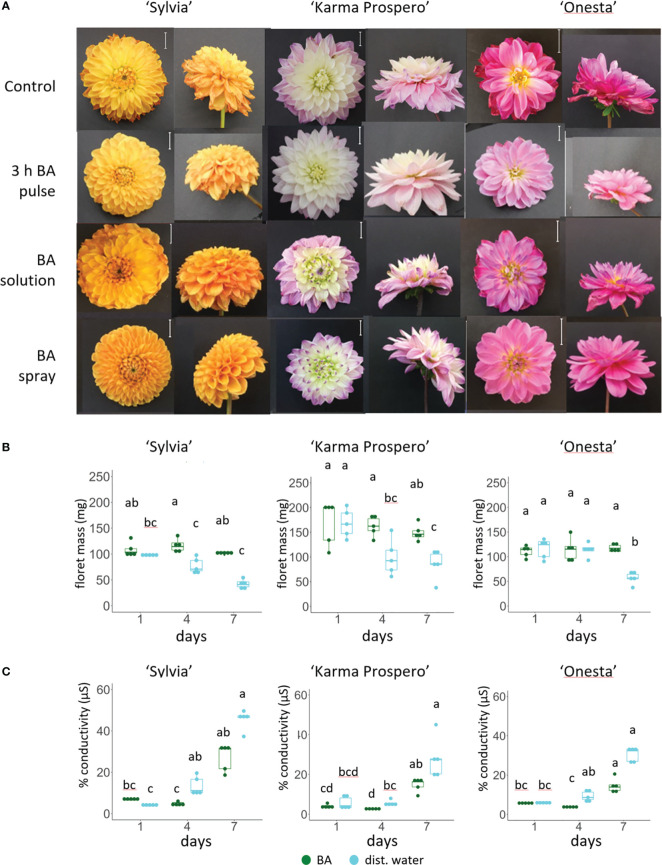
Effect of exogenous cytokinin on floret senescence in dahlia cv.s Sylvia, Karma Prospero and Onesta. Stems were held in distilled water (control), compared to stems treated with cytokinin (BA) applied as a pulse (100 µM), as a solution (100 µM), or a spray (100 µM). **(A)** Flower head appearance 7 days after harvesting at Stage III; scale bars represent 20 mm); **(B)** floret mass **(C)** ion leakage, 1, 4 and 7 days after cutting stage (n=5). Different letters indicate significant differences *p* < 0.05, based on a 2-way ANOVA followed by a Tukey’s test or a Kruskall Wallis test followed by a Dunn’s *post hoc* test if the data did not fit the normality and equal variance criteria required.

RNAseq showed that expression of cytokinin signaling genes also changed significantly during floret senescence, hence exogenous application of cytokinin was tested. Cytokinin (BA) application as a spray had a dramatic effect in delaying visible signs of floret wilting after 7 days in distilled water (**Figure 10A**) in all three cultivars, ‘Sylvia’, ‘Karma Prospero’ and ‘Onesta’. In contrast, continuous addition of BA to the vase water accelerated senescence. A pulse of BA for 3h just after harvest appeared to have an intermediate effect with differential effects in the different cultivars. Wilting was inhibited in flowers of all three cultivars, though in ‘Sylvia’ there was some wilting after 7 days and ‘Onesta’ spray- treated flowers opened significantly better compared with pulsed flowers. The effects of BA spray treatment were mirrored by a delay in the reduction of fresh weight during vase life. This effect was more pronounced in ‘Sylvia’ and ‘Karma Prospero’, where fresh weight was significantly higher than in controls both after 4 days and 7 days of vase life. There was an interaction between time and treatment in relation to mass change for all three cultivars (*p* < 0.05). In ‘Onesta’ a significant 2-fold difference (*p* < 0.05) in floret mass between BA sprayed and control flowers was only seen after 7 days (**Figure 10B**). There was an interaction between time and treatment for ion leakage in both ‘Sylvia’ and ‘Onesta’ florets (p < 0.01) but not ‘Karma Prospero’. There was a significant over 2-fold (*p* < 0.05) reduction in ion leakage by the BA spray treatment in all three cultivars after 4 days of vase life, while after 7 days although there was a reduction it was not significant (**Figure 10C**).

### 3.9 Ethylene and cytokinin-related gene expression in response to exogenous treatments

**Figure 11:**
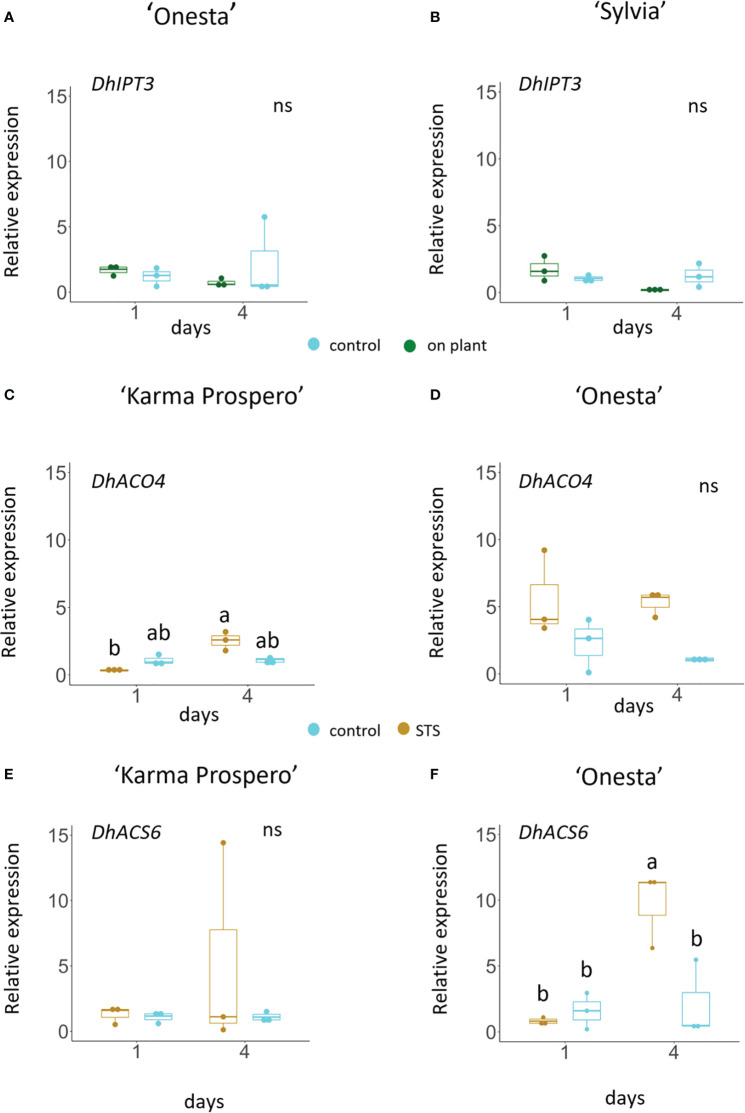
Relative gene expression (by RT-qPCR) in dahlia florets 1 and 4 days after harvest at Stage III: *Dp IPT3* **(A, C)** *DpACO4* **(C, D)** and *DpACS6* **(E, F)** in ‘Onesta’ **(A, D, F)** Sylvia’ **(B)** ‘Karma Prospero’ **(C, E)** from flowers treated as controls (distilled water) or compared to flowers left on the plant **(A, B)** a 1 h pulse of 4 mM STS **(C–F)**, using β-tubulin as a reference (n=3). Different letters indicate significant differences (*p* < 0.05) amongst the four samples for each panel based on ANOVA followed by a Tukey’s test.

Based on the transcriptome sequences from the ‘Sylvia’ florets, the expression of key genes related to ethylene and cytokinin signalling could be explored across different dahlia cultivars in response to cutting from the plant and exogenous treatments (**Figure 11**) using RT-qPCR. Expression of *DpIPT3*, involved in cytokinin biosynthesis showed an interaction between treatment and time in ‘Sylvia’ (*p* <0.05) but not in ‘Onesta’. Expression fell slightly in mid-whorl florets from flowers sampled at stage III both in ‘Onesta’ and ‘Sylvia’ between days 1 and 4 when on the plant, although due to the variability the difference was not statistically significant. There was no difference in expression in cut flowers (**Figures 11A, B**).

Changes in the expression of ethylene biosynthesis genes *DpACO4* and *DpACS6* when cut flowers were treated with STS, differed between cultivars. In cv. ‘Karma Prospero’ *DpACO4* expression showed an interaction between treatment and time (p < 0.01). Expression was significantly lower in STS treated flowers (*p* < 0.05) 1 day after treatment, compared to day 4 (**Figure 11C**), but did not increase in untreated flowers. In ‘Onesta’ florets expression of *DpACO4* showed no interaction between time and treatment. Expression in STS treated flowers appeared to be higher compared to controls on both days, but changes were not statistically significant, again probably due to the variability across replicates. (**Figure 11D**). *DpACS6* expression showed no consistent difference between groups on either day in ‘Karma Prospero’ (**Figure 11E**). However, in cv. ‘Onesta’ *DpACS6* expression was significantly (*p* < 0.05) over 4-fold higher 4 days after treatment in STS treated flowers compared to controls at day 4 and 12-fold higher than STS treated flowers at day 1 (*p* < 0.05), there was no significant difference between controls after 1 or 4 days (**Figure 11F**).

## 4 Discussion

In this work dahlia floret senescence was compared both on and off the plant, and across different varieties; transcriptomic analysis was used to assess changes in gene expression across a single flower head and between flower heads of different ages. Firstly, differences between senescence on and off the plant was investigated. Loss of mass seen here is an early sign of floral senescence in cut flowers of other dahlia cultivars, e.g. ‘Kokucho’ (Shimizu-Yumoto and Ichimura, 2013) and in flowers of other species e.g. lilies (Battelli et al., 2011), especially in those where petals or florets do not abscise turgid (van Doorn and Woltering, 2008; Rogers and Stead, 2011). A rise in conductivity is a widely accepted symptom of petal senescence (Whitlow et al., 1992), and the later rise in conductivity is also in line with other flowers such as rose (Torre et al., 1999) and lily (Lombardi et al., 2015). Detachment from the plant significantly accelerated both processes. This comparison has not been reported previously for dahlia or many other flowers, but in lilies senescence progression was also accelerated by cutting from the plant and was associated with changes in the balance of growth regulators (Arrom and Munne-Bosch, 2012). The significant loss of mass in ‘Karma Prospero’ after 4 days of vase life without an increase in conductivity may reflect a loss of water that is not yet accompanied by an increase in membrane damage. ‘Onesta’ senescence appeared to progress more slowly, indicating cultivar-related variation. Of interest is also the slight rise in conductivity at day 4 in on plant flowers of all three cultivars. This may be due to the continued development on plant, and is worthy of further investigation.

The RNA-sequencing revealed interesting differences in the changes in expression between florets at the same position in flower heads of increasing age, compared to those across a single flower head. The larger number of DEGs in the latter comparison is consistent with most of the changes being floret-dependent rather than related to ageing of the whole head. This confirms the similarity of senescence in composite flowers to senescence in cyme inflorescences e.g. in Arabidopsis (Wagstaff et al., 2009) or wallflower (Price et al., 2008; Mohd Salleh et al., 2016). The down regulation of *DpSAG12* in outer florets compared to inner florets is perhaps surprising as this gene is a widely accepted senescence marker (Macnish et al., 2010). However, this may indicate that even the outer florets analysed here are at a relatively early stage of senescence. The up regulation of other SAG genes such as *DpSAG13, DpSAG14*, and *DpSAG21*, is consistent in that all these genes are expressed earlier, at least in leaf senescence (Weaver et al., 1998). Several senescence-related genes are linked with ROS responses (*DpETFALPHA, DpSAG21* and *DpSAG13*), consistent with other studies on petal senescence (Rogers and Munné-Bosch, 2016), indicating that in dahlia too ROS may participate in floral senescence regulation. The up regulation of a number of cell death-related genes is consistent with other studies showing that cell death starts early in the mesophyll even in petals that do not show signs of senescence (Wagstaff et al., 2003; Wang et al., 2021). VPEs and metacaspases increase in expression in senescent petals of other many other species (Rogers, 2013). The RNAseq showed strong down regulation of two dahlia VPE genes in the comparison of young florets across flower heads, but up regulation of all the VPE genes when comparing older and younger florets in the same head again indicating differences between these two developmental steps. The RT-qPCR was not able to confirm this pattern although there was a slight rise in expression in SIV-out florets compared to the other two stages and it was only possible to verify the expression of one dahlia gene. This inconsistency might be due to the complexity of the dahlia genome resulting in lack of complete primer specificity which is difficult to verify without a genome sequence. VPEs are required for some forms of plant PCD, have caspase activity (Yamada et al., 2019), and their expression increases with petal senescence in several species (e.g. lily, Battelli et al., 2011; *Ipomea*, Yamada et al., 2009). This suggests that despite the lack of *DpSAG12* up regulation, some late senescence processes are starting to be activated in outer SIV florets.

The up regulation of NAC and WRKY TF families in the SIII-in vs. SIV-in comparison suggests that even florets collected from the same position on the flower head are already aging as the whole flower head ages, before any visible signs of senescence. WRKY6 has been found to positively mediate leaf senescence in *Arabidopsis thaliana*, and be highly up regulated in floral abscission zones, (Robatzek and Somssich, 2002), whilst WRKY4 has been implicated in plant stress responses (Lai et al., 2008). Many MYB transcription factors also increased with floret age. *MYB108*, up regulated in SIV-out vs. SIV-in florets is involved in the interplay between ethylene and jasmonic acid in rose, and when silenced petal senescence was delayed (Zhang et al., 2019). RT-qPCR validated the RNAseq data for all three TFs tested.

In cv. ‘Sylvia’ the transcriptome analysis showed up regulation with floret senescence both of ACC synthase, which encodes the rate limiting enzyme of ethylene biosynthesis (Yang & Hoffman, 1984), as well as downstream components of the signal transduction pathway. These included a large number of ERF transcription factors with possible gene interactions to senescence-related genes such as WRKY transcription factors and vacuolar processing enzymes, two of which were validated by RT-qPCR. This indicates the activation of ethylene pathways during individual floret senescence. However, there were very few changes in ERF family transcription factor expression in the SIII-in vs. SIV-in comparison. This indicates that ethylene signalling changes across the head rather than with increasing head age. This is supported by the off-plant experiments where there were different effects of STS on overall flower head appearance compared to changes in senescence markers in individual florets of the same whorl after different periods post-harvest.

Across cultivars, responses to ethylene-related treatments differed. Unlike in ‘Karma Thalia’ (Dole et al., 2009) exogenous ethylene *via* CEPA accelerated weight loss in all three cultivars in line with previous findings in cv. Kokucho (Shimizu-Yumoto and Ichimura, 2013). However, in this study, effects of STS varied amongst cultivars, having a strong effect in delaying symptoms of senescence in ‘Sylvia’ and ‘Karma Prospero’ but not in ‘Onesta’. This suggests variability in the role of endogenous ethylene across cultivars, as in ‘Kokucho’ endogenous ethylene was produced, albeit at low levels throughout floret senescence (Shimizu-Yumoto and Ichimura, 2013). Indeed, there were also differences in the effect of STS treatment on expression of ethylene biosynthetic genes *DpACO4* and *DpACS6* between ‘Karma Prospero’ and ‘Onesta’. This suggests a response to inhibition of endogenous ethylene signalling even in ‘Onesta’, despite the lack of visual effects on senescence progression. The up regulation of ethylene biosynthesis genes in response to STS contrasts with rose where STS reduced expression of at least some *ACS* and *ACO* gene family members (Ma et al., 2005), however the regulation of the expression of both these gene families is complex (de Azevedo Souza et al., 2008; Houben and Van de Poel, 2019). Although STS reduced deterioration in overall visual appearance in ‘Karma Prospero’ flower heads, ion leakage in mid whorl florets was not significantly affected. Both *DpERF1/2* that activate downstream ethylene-responsive genes, and *DpEBF1/2* that act as negative regulators of ethylene signalling (Li and Guo, 2007) were up regulated in older florets (based on the transcriptome analysis and validated by RT-qPCR). This suggests a delicate balance of ethylene signalling during dahlia floret senescence. However, *DpMAPK6*, that also activates ethylene biosynthesis (Xu et al., 2008) was not up regulated between SIV and SIII inner florets, whereas it was up regulated in SIV outer compared to inner florets. This suggests that although some of the regulatory pathway is already activated in younger florets, other steps are only activated in the older outer florets.

Treatment with a pulse or spray of BA consistently improved flower appearance, floret mass and cellular membrane integrity. This agrees with other studies, where spraying whole dahlia flowers with BA (50 µM) increased their vase life (Shimizu-Yumoto & Ichimura, 2013). However, flowers treated with a 100 µM solution of BA showed severe wilting compared to flowers treated with a pulse or spray of BA. This effect may be due to the induction of hypersensitivity to cytokinins, as high concentrations of cytokinins have been found to induce PCD in both carrot and Arabidopsis (Carimi et al., 2003). The role of cytokinins as endogenous regulators of dahlia floret senescence is supported by the down regulation of IPT shown by the transcriptomic analysis and up regulation of cytokinin oxidase in older florets, before any visible signs of senescence, the latter validated by RT-qPCR. A fall in cytokinin content with flower senescence has been noted in many other flowers (e.g. rose, Mayak and Halevy, 1970) as has a rise in cytokinin oxidase expression in senescing carnation petals (Hoeberichts et al., 2007). The fall in IPT expression with floret age is indicated by real time PCR results on plant, both in ‘Sylvia’ and ‘Onesta’ although likely due to variability amongst replicates the differences were not statistically significant. When flowers were detached, expression of this cytokinin biosynthesis gene expression appeared more stable. This consistent difference across cultivars may be important, explaining the more rapid senescence in cut flowers. It may be caused by a more rapid loss of cytokinins triggering compensating biosynthesis. The up regulation of *DpARR* genes seen in ageing dahlia florets was previously noted in senescent Arabidopsis petals (Wagstaff et al., 2009). This may be associated with increased sensitivity to the reducing levels of cytokinin, needed to keep the tissue functional during remobilisation, and may also explain the increase in cytokinin receptor *DpCRE1* expression. The up regulation of both A-type negative regulators of cytokinin signalling and positive B-type ARRs in outer compared to inner SIV florets is perhaps surprising but may be necessary for maintaining sufficient cytokinin signalling during senescence. A reduction in expression of cytokinin signalling genes may only occur at more advanced stages of senescence than sampled here.

In conclusion, the data show that regulation of cytokinin biosynthesis may be an important factor in senescence of cut flowers compared to those on the plant. The role of ethylene as a senescence regulator varies across dahlia cultivars and both cytokinin and ethylene signalling are under the complex control of both positive and negative regulators as the florets senesce. The RNAseq data provide a wealth of new targets for further validation but also indicate underlying patterns in floret senescence in complex flower heads. Floret position in the flower head appears to be critical to its senescence programme and indeed outer florets of older flower heads show changes in gene expression compared to inner florets. This is consistent with activation of senescence and cell death processes several days before visual senescence. However, the up regulation of senescence-associated transcription factors indicates that even in inner florets, the ageing of the head is inducing the initial stages of senescence activation in older flowers, but not yet cell death. A better understanding of how senescence is regulated in composite flowers may help in identifying gene targets for breeding, and pathways that may lead to new improved treatments to extend vase life and reduce waste.

## Data availability statement

The datasets presented in this study can be found in online repositories. The names of the repository/repositories and accession number(s) can be found below: https://www.ncbi.nlm.nih.gov/ , PRJNA742864.

## Author contributions

MC, AC and AB conducted the experimental work and drafted the manuscript, BL and IM assisted with data analysis, HR and AS designed the project and co-wrote the manuscript. All authors revised the manuscript. All authors contributed to the article and approved the submitted version.
