# Supplementary material for: Senescence in dahlia flowers is regulated by a complex interplay between flower age and floret position
Source: Front Plant Sci. 2023 Jan 13;13:1085933. doi: 10.3389/fpls.2022.1085933 (PMC9880482; doi:10.3389/fpls.2022.1085933)
Supplement: Supplementary file 1 [file DataSheet_1.zip › Supplementary Table S1 - list of primers for real time PCR.docx]

**Supplementary Table S1 – all primers used for real time PCR**

|  | Forward | Reverse |
| --- | --- | --- |
| *DhIPT3* | TCGGCATTCTTCTTCGGCTA | AGTGTTCGTGATAGGTGCCA |
| *DhACO4* | GAGAGAGATGTCACGATGGA | CCTCTGCTCCATACACTTCT |
| *DhACS6* | CATGAACCAAGTACGAGGAA | ACTCCAGTTCGCCATCTCAA |
| *Dhβ-tubulin* | GAGTCTCCGGTGTACTTGCC | CGCCCTGGATGTGAAGGATT |
