# Supplementary material for: Senescence in dahlia flowers is regulated by a complex interplay between flower age and floret position
Source: Front Plant Sci. 2023 Jan 13;13:1085933. doi: 10.3389/fpls.2022.1085933 (PMC9880482; doi:10.3389/fpls.2022.1085933)
Supplement: Supplementary file 1 [file DataSheet_1.zip › Supplementary Table S3- ethylene related gene interaction lists.docx]

**Supplementary Table S3. List of genes in RESPONSE TO ETHYLENE GO:0009723 identified as interactors in Cytoscape**

**SIII-in vs. SIV-in up-regulated**

| Query term* | Gene name | Function |
| --- | --- | --- |
| **AT4G11280**  **AT4G34410**  attribute  attribute  attribute  attribute  **AT4G37260**  **AT4G32940**  **AT1G13960**  **AT1G62300**  **AT5G47220**  attribute  attribute  attribute  attribute  attribute  attribute  attribute  attribute  attribute  **AT5G25350**  attribute  attribute  attribute  attribute  attribute  **AT5G13910**  **AT5G18560**  attribute  **AT3G51550**  attribute  **AT5G40990** | **ACS6**  ERF109  ERF13  ERF1A  WRKY33  ERF6  MYB73  GAMMA-VPE  WRKY4  WRKY6  ERF2  ERF4  CYP94C1  ERF15  ERF018  ERF094  WRKY40  NAC102  ERF105  ERF104  EBF2  dVPE  AT1G08750  WRKY18  AT2G34600  WRKY38  LEP  ERF086  bVPE  FER  ALPHA-VPE  GLIP1 | **1-aminocyclopropane-1-carboxylate synthase 6** [Source:UniProtKB/Swiss-Prot;Acc:Q9SAR0]  Ethylene-responsive transcription factor ERF109 [Source:UniProtKB/Swiss-Prot;Acc:Q9SZ06]  Ethylene-responsive transcription factor 13 [Source:UniProtKB/Swiss-Prot;Acc:Q8L9K1]  Ethylene-responsive transcription factor 1A [Source:UniProtKB/Swiss-Prot;Acc:O80337]  Probable WRKY transcription factor 33 [Source:UniProtKB/Swiss-Prot;Acc:Q8S8P5]  Ethylene-responsive transcription factor 6 [Source:UniProtKB/Swiss-Prot;Acc:Q8VZ91]  myb domain protein 73 [Source:TAIR;Acc:AT4G37260]  Vacuolar-processing enzyme gamma-isozyme [Source:UniProtKB/Swiss-Prot;Acc:Q39119]  Probable WRKY transcription factor 4 [Source:UniProtKB/Swiss-Prot;Acc:Q9XI90]  WRKY transcription factor 6 [Source:UniProtKB/Swiss-Prot;Acc:Q9C519]  Ethylene-responsive transcription factor 2 [Source:UniProtKB/Swiss-Prot;Acc:O80338]  Ethylene-responsive transcription factor 4 [Source:UniProtKB/Swiss-Prot;Acc:O80340]  Cytochrome P450 94C1 [Source:UniProtKB/Swiss-Prot;Acc:Q9ZUX1]  Ethylene-responsive transcription factor 15 [Source:UniProtKB/Swiss-Prot;Acc:Q8VYM0]  Ethylene-responsive transcription factor ERF018 [Source:UniProtKB/Swiss-Prot;Acc:Q9S7L5]  Ethylene-responsive transcription factor ERF094 [Source:UniProtKB/Swiss-Prot;Acc:Q9LND1]  Probable WRKY transcription factor 40 [Source:UniProtKB/Swiss-Prot;Acc:Q9SAH7]  NAC domain-containing protein 102 [Source:UniProtKB/Swiss-Prot;Acc:Q8H115]  Ethylene-responsive transcription factor ERF105 [Source:UniProtKB/Swiss-Prot;Acc:Q8VY90]  Ethylene-responsive transcription factor ERF104 [Source:UniProtKB/Swiss-Prot;Acc:Q9FKG1]  EIN3-binding F-box protein 2 [Source:UniProtKB/Swiss-Prot;Acc:Q708Y0]  Vacuolar-processing enzyme delta-isozyme [Source:UniProtKB/Swiss-Prot;Acc:Q9LJX8]  Peptidase C13 family [Source:TAIR;Acc:AT1G08750]  WRKY transcription factor 18 [Source:UniProtKB/Swiss-Prot;Acc:Q9C5T4]  Protein TIFY 5B [Source:UniProtKB/Swiss-Prot;Acc:O64687]  Probable WRKY transcription factor 38 [Source:UniProtKB/Swiss-Prot;Acc:Q8GWF1]  Ethylene-responsive transcription factor LEP [Source:UniProtKB/Swiss-Prot;Acc:Q9M644]  Ethylene-responsive transcription factor ERF086 [Source:UniProtKB/Swiss-Prot;Acc:Q6J9Q2]  Vacuolar-processing enzyme beta-isozyme [Source:UniProtKB/Swiss-Prot;Acc:Q39044]  Receptor-like protein kinase FERONIA [Source:UniProtKB/Swiss-Prot;Acc:Q9SCZ4]  Vacuolar-processing enzyme alpha-isozyme [Source:UniProtKB/Swiss-Prot;Acc:P49047]  GDSL esterase/lipase 1 [Source:UniProtKB/Swiss-Prot;Acc:Q9FLN0] |

*ATG codes in bold are genes identified in the dahlia DEGs; Attribute: are genes identified within Cytoscape as interactors

**SIV-in vs. SIV-out up-regulated**

| Query term* | Gene name | Function |
| --- | --- | --- |
| **AT3G23250.1**  **AT3G23240.1**  attribute  **AT2G44840.1**  attribute  **AT5G47230.1**  attribute  attribute  attribute  **AT3G12500.1**  **AT5G25350.1**  **AT2G38120.1**  attribute  attribute  attribute  attribute  attribute  **AT1G28370.1**  attribute  attribute  **AT5G47220.1**  attribute  **AT4G05100.1**  **AT2G43790.1**  **AT1G15520.1**  attribute  attribute  **AT3G24500.1**  attribute  **AT2G47190.1**  **AT1G53170.1**  attribute  attribute  **AT1G06180.1**  **AT2G25490.1**  **AT5G44790.1**  **AT5G67300.1**  attribute  **AT3G16770.1**  **AT3G23230.1**  **AT5G47390.1**  **AT3G06490.1**  attribute  **AT4G34460.2**  **AT5G40990.1**  **AT1G66350.1** | MYB15  ERF1B  ERF1A  ERF13  ERF6  ERF5  ERF109  AT2G43590  HEL  CHI-B  EBF2  AUX1  CYN  MBF1B  DREB1B  ERF104  ERF018  ERF11  ERF105  ERF017  ERF2  CHI  AtMYB74  MPK6  ABCG40  ERF4  AT2G43580  MBF1C  AT2G43620  ATMYB2  ERF8  MBF1A  ERF019  ATMYB13  EBF1  AT5G44790.1  MYB44  ERF014  RAP2-3  ERF098  AT5G47390.1  MYB108  ERF022  GB1  GLIP1  RGL1 | myb domain protein 15 [Source:TAIR;Acc:AT3G23250]  Ethylene-responsive transcription factor 1B [Source:UniProtKB/Swiss-Prot;Acc:Q8LDC8]  Ethylene-responsive transcription factor 1A [Source:UniProtKB/Swiss-Prot;Acc:O80337]  Ethylene-responsive transcription factor 13 [Source:UniProtKB/Swiss-Prot;Acc:Q8L9K1]  Ethylene-responsive transcription factor 6 [Source:UniProtKB/Swiss-Prot;Acc:Q8VZ91]  Ethylene-responsive transcription factor 5 [Source:UniProtKB/Swiss-Prot;Acc:O80341]  Ethylene-responsive transcription factor ERF109 [Source:UniProtKB/Swiss-Prot;Acc:Q9SZ06]  Chitinase family protein [Source:TAIR;Acc:AT2G43590]  Hevein-like preproprotein [Source:UniProtKB/Swiss-Prot;Acc:P43082]  Basic endochitinase B [Source:UniProtKB/Swiss-Prot;Acc:P19171]  EIN3-binding F-box protein 2 [Source:UniProtKB/Swiss-Prot;Acc:Q708Y0]  Auxin transporter protein 1 [Source:UniProtKB/Swiss-Prot;Acc:Q96247]  Cyanate hydratase [Source:UniProtKB/Swiss-Prot;Acc:O22683]  Multiprotein-bridging factor 1b [Source:UniProtKB/Swiss-Prot;Acc:Q9LXT3]  Dehydration-responsive element-binding protein 1B [Source:UniProtKB/Swiss-Prot;Acc:P93835]  Ethylene-responsive transcription factor ERF104 [Source:UniProtKB/Swiss-Prot;Acc:Q9FKG1]  Ethylene-responsive transcription factor ERF018 [Source:UniProtKB/Swiss-Prot;Acc:Q9S7L5]  Ethylene-responsive transcription factor 11 [Source:UniProtKB/Swiss-Prot;Acc:Q9C5I3]  Ethylene-responsive transcription factor ERF105 [Source:UniProtKB/Swiss-Prot;Acc:Q8VY90]  Ethylene-responsive transcription factor ERF017 [Source:UniProtKB/Swiss-Prot;Acc:Q84QC2]  Ethylene-responsive transcription factor 2 [Source:UniProtKB/Swiss-Prot;Acc:O80338]  chitinase, putative [Source:TAIR;Acc:AT2G43570]  myb domain protein 74 [Source:TAIR;Acc:AT4G05100]  Mitogen-activated protein kinase 6 [Source:UniProtKB/Swiss-Prot;Acc:Q39026]  ABC transporter G family member 40 [Source:UniProtKB/Swiss-Prot;Acc:Q9M9E1]  Ethylene-responsive transcription factor 4 [Source:UniProtKB/Swiss-Prot;Acc:O80340]  Chitinase family protein [Source:TAIR;Acc:AT2G43580]  Multiprotein-bridging factor 1c [Source:UniProtKB/Swiss-Prot;Acc:Q9LV58]  Chitinase family protein [Source:TAIR;Acc:AT2G43620]  myb domain protein 2 [Source:TAIR;Acc:AT2G47190]  Ethylene-responsive transcription factor 8 [Source:UniProtKB/Swiss-Prot;Acc:Q9MAI5]  Multiprotein-bridging factor 1a [Source:UniProtKB/Swiss-Prot;Acc:Q9SJI8]  Ethylene-responsive transcription factor ERF019 [Source:UniProtKB/Swiss-Prot;Acc:O80542]  myb domain protein 13 [Source:TAIR;Acc:AT1G06180]  EIN3-binding F-box protein 1 [Source:UniProtKB/Swiss-Prot;Acc:Q9SKK0]  Copper-transporting ATPase RAN1 [Source:UniProtKB/Swiss-Prot;Acc:Q9S7J8]  Transcription factor MYB44 [Source:UniProtKB/Swiss-Prot;Acc:Q9FDW1]  Ethylene-responsive transcription factor ERF014 [Source:UniProtKB/Swiss-Prot;Acc:Q9LPE8]  Ethylene-responsive transcription factor RAP2-3 [Source:UniProtKB/Swiss-Prot;Acc:P42736]  Ethylene-responsive transcription factor ERF098 [Source:UniProtKB/Swiss-Prot;Acc:Q9LTC5]  myb-like transcription factor family protein [Source:TAIR;Acc:AT5G47390]  Transcription factor MYB108 [Source:UniProtKB/Swiss-Prot;Acc:Q9LDE1]  Ethylene-responsive transcription factor ERF022 [Source:UniProtKB/Swiss-Prot;Acc:Q9LQ28]  GDSL esterase/lipase 1 [Source:UniProtKB/Swiss-Prot;Acc:Q9FLN0]  DELLA protein RGL1 [Source:UniProtKB/Swiss-Prot;Acc:Q9C8Y3] |

*ATG codes in bold are genes identified in the dahlia DEGs; Attribute: are genes identified within Cytoscape as interactors

**SIV-in vs. SIV-out up-regulated**

| Query term* | Gene name | Function |
| --- | --- | --- |
| **AT3G23250.1**  **AT3G23240.1**  attribute  **AT2G44840.1**  attribute  **AT3G12500.1**  attribute  attribute  attribute  attribute  attribute  attribute  **AT5G47220.1**  attribute  attribute  attribute  attribute  attribute  **AT2G43790.1**  **AT5G25350.1**  **AT1G15520.1**  **AT3G24500.1**  attribute  attribute  attribute  attribute  **AT2G25490.1**  attribute  **AT5G44790.1**  **AT3G16770.1**  attribute  **AT3G23230.1**  **AT3G06490.1**  attribute  **AT4G34460.2**  **AT5G40990.1**  **AT2G31180.1** | MYB15  ERF1B  ERF1A  ERF13  HEL  CHI-B  AT2G43590  ERF6  ERF109  CYN  MBF1B  ERF018  ERF2  ERF11  ATEP3  CHI  AT2G43610  ERF094  MPK6  EBF2  ABCG40  MBF1C  AT2G43580  AT2G43620  MBF1A  RAP2-2  EBF1  ERF019  AT5G44790.1  RAP2-3  ERF014  ERF098  MYB108  ERF022  GB1  GLIP1  ATMYB14 | myb domain protein 15 [Source:TAIR;Acc:AT3G23250]  Ethylene-responsive transcription factor 1B [Source:UniProtKB/Swiss-Prot;Acc:Q8LDC8]  Ethylene-responsive transcription factor 1A [Source:UniProtKB/Swiss-Prot;Acc:O80337]  Ethylene-responsive transcription factor 13 [Source:UniProtKB/Swiss-Prot;Acc:Q8L9K1]  Hevein-like preproprotein [Source:UniProtKB/Swiss-Prot;Acc:P43082]  Basic endochitinase B [Source:UniProtKB/Swiss-Prot;Acc:P19171]  Chitinase family protein [Source:TAIR;Acc:AT2G43590]  Ethylene-responsive transcription factor 6 [Source:UniProtKB/Swiss-Prot;Acc:Q8VZ91]  Ethylene-responsive transcription factor ERF109 [Source:UniProtKB/Swiss-Prot;Acc:Q9SZ06]  Cyanate hydratase [Source:UniProtKB/Swiss-Prot;Acc:O22683]  Multiprotein-bridging factor 1b [Source:UniProtKB/Swiss-Prot;Acc:Q9LXT3]  Ethylene-responsive transcription factor ERF018 [Source:UniProtKB/Swiss-Prot;Acc:Q9S7L5]  Ethylene-responsive transcription factor 2 [Source:UniProtKB/Swiss-Prot;Acc:O80338]  Ethylene-responsive transcription factor 11 [Source:UniProtKB/Swiss-Prot;Acc:Q9C5I3]  homolog of carrot EP3-3 chitinase [Source:TAIR;Acc:AT3G54420]  chitinase, putative [Source:TAIR;Acc:AT2G43570]  Chitinase family protein [Source:TAIR;Acc:AT2G43610]  Ethylene-responsive transcription factor ERF094 [Source:UniProtKB/Swiss-Prot;Acc:Q9LND1]  Mitogen-activated protein kinase 6 [Source:UniProtKB/Swiss-Prot;Acc:Q39026]  EIN3-binding F-box protein 2 [Source:UniProtKB/Swiss-Prot;Acc:Q708Y0]  ABC transporter G family member 40 [Source:UniProtKB/Swiss-Prot;Acc:Q9M9E1]  Multiprotein-bridging factor 1c [Source:UniProtKB/Swiss-Prot;Acc:Q9LV58]  Chitinase family protein [Source:TAIR;Acc:AT2G43580]  Chitinase family protein [Source:TAIR;Acc:AT2G43620]  Multiprotein-bridging factor 1a [Source:UniProtKB/Swiss-Prot;Acc:Q9SJI8]  Ethylene-responsive transcription factor RAP2-2 [Source:UniProtKB/Swiss-Prot;Acc:Q9LUM4]  EIN3-binding F-box protein 1 [Source:UniProtKB/Swiss-Prot;Acc:Q9SKK0]  Ethylene-responsive transcription factor ERF019 [Source:UniProtKB/Swiss-Prot;Acc:O80542]  Copper-transporting ATPase RAN1 [Source:UniProtKB/Swiss-Prot;Acc:Q9S7J8]  Ethylene-responsive transcription factor RAP2-3 [Source:UniProtKB/Swiss-Prot;Acc:P42736]  Ethylene-responsive transcription factor ERF014 [Source:UniProtKB/Swiss-Prot;Acc:Q9LPE8]  Ethylene-responsive transcription factor ERF098 [Source:UniProtKB/Swiss-Prot;Acc:Q9LTC5]  Transcription factor MYB108 [Source:UniProtKB/Swiss-Prot;Acc:Q9LDE1]  Ethylene-responsive transcription factor ERF022 [Source:UniProtKB/Swiss-Prot;Acc:Q9LQ28]  Guanine nucleotide-binding protein subunit beta [Source:UniProtKB/Swiss-Prot;Acc:P49177]  GDSL esterase/lipase 1 [Source:UniProtKB/Swiss-Prot;Acc:Q9FLN0]  myb domain protein 14 [Source:TAIR;Acc:AT2G31180] |

*ATG codes in bold are genes identified in the dahlia DEGs; Attribute: are genes identified within Cytoscape as interactors
