# Supplementary figures and images for: Senescence in dahlia flowers is regulated by a complex interplay between flower age and floret position
Source: Front Plant Sci. 2023 Jan 13;13:1085933. doi: 10.3389/fpls.2022.1085933 (PMC9880482; doi:10.3389/fpls.2022.1085933)

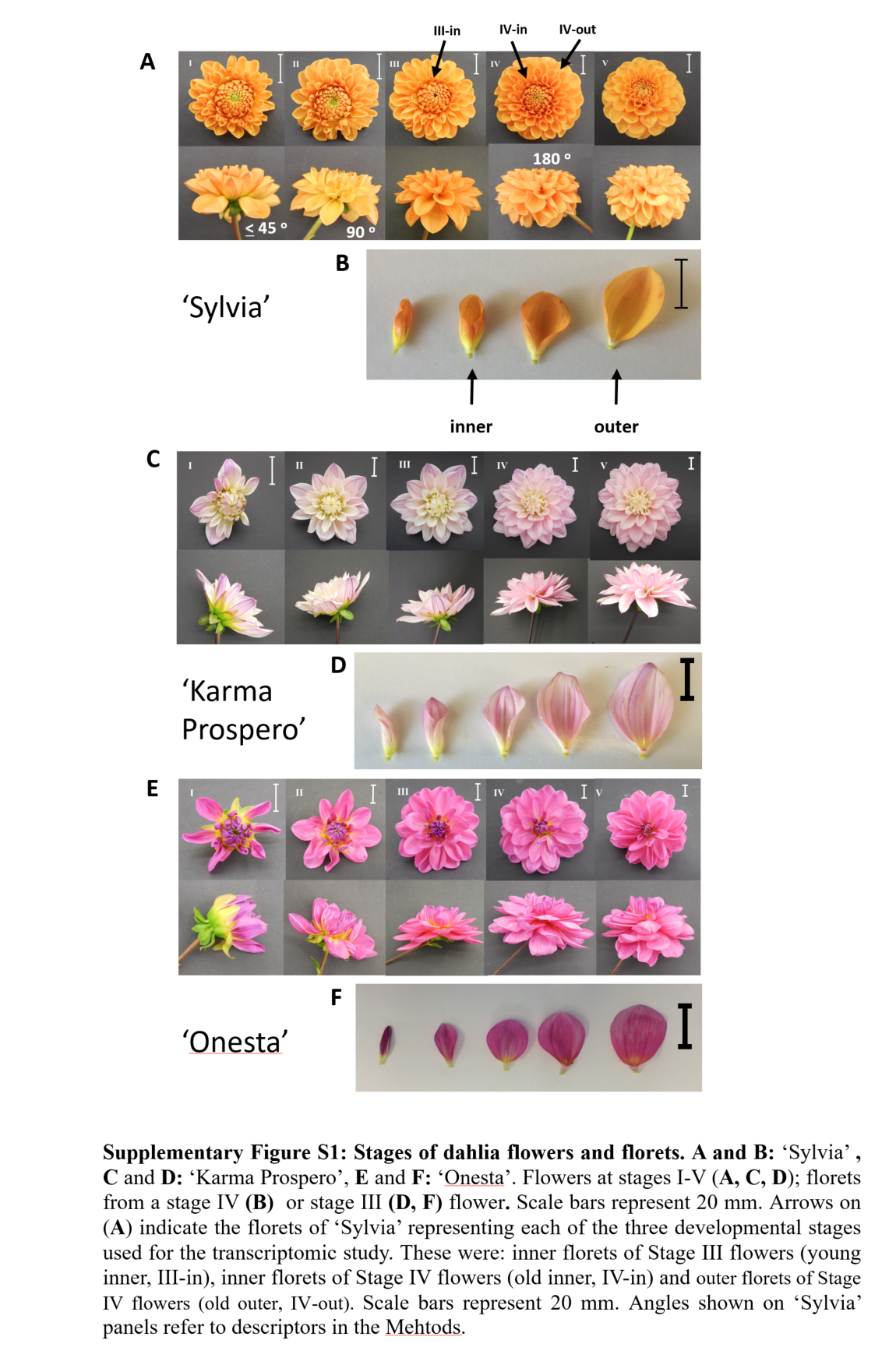

Supplement: Supplementary file 1 [file DataSheet_1.zip › SUPPLEMENTARY FIG 1 REVISED.tiff]
